# Working Dog Service, Harmful Agent Exposure and Decontamination

**DOI:** 10.3389/fvets.2022.892998

**Published:** 2022-05-02

**Authors:** Carla L. Jarrett, Morgan Brathwaite, Robert M. Gogal, Steven D. Holladay

**Affiliations:** Department of Biomedical Sciences, College of Veterinary Medicine, University of Georgia, Athens, GA, United States

**Keywords:** working dogs, military, search and rescue (SAR), chemical warfare (CW) agent, personal protective equipment, decontamination

## Abstract

Working dogs are widely used by service professionals and the military for diverse roles that include sentry, patrol, messenger, tracking, search and rescue, law enforcement, apprehension, as well as explosives and narcotics detection. The expected tasks performed are in many ways determined by the breed, which is customarily a German Shepherd, Dutch Shepherd, Golden Retriever, Border Collie, Labrador Retriever, Beagle, or Belgium Malinois. Working dogs may be subject to injury from dangerous work environments or harmful agent exposure. Personal protective equipment (PPE) has been developed for such dogs, but may impede performance of duties or be poorly tolerated. Canine-specific field-use ready decontamination techniques and kits are therefore needed for use on working dogs that have encountered a harmful agent exposure. This report briefly reviews the development of the military working dog and examines personal protective equipment and decontamination techniques for working dogs after exposure to harmful biologic or chemical agents.

## History

The French utilized dogs to guard naval installations and docks in the early 14th century and later employed dogs to control gangs in Paris in 1895; the Germans similarly used dogs in 1896 (1). Several European countries, following the lead of Germany, also began to use dogs during World War I (WWI). During this time both the Allies and the Central Powers developed gas masks and chemical protective suits for these highly regarded animals (2).

In 1920, a first-of-its-kind canine training school was established at Greenheide, Germany where future police dogs learned basic obedience, tracking, and searching (1). Police dog training continued to spread throughout the globe and with the advent of World War II (WWII) many countries utilized their police dogs for the war effort. In 1942, the United States (US) recognized the need to expand the use of dogs for military service and began accepting dogs trained by civilian programs, a step which ultimately led to the establishment of the US Army Quartermaster Corps, which along with the US Army Veterinary Corps developed a screening and selection process for the thousands of private citizen dogs that were donated to the Dogs for Defense program (2). Breed, size, age, temperament and health criteria were established, and dogs that qualified were matched with handlers and the teams were trained in different specialties (2). During WWII the trained teams were assigned to Army and Marine units, where these dogs assisted by performing sentry, messenger, scout, and both enemy and mine detection duties (2, 3). During the Korean War in 1951, the Army dog program was shifted from the Quartermaster Corps to the Military Police Corps, which trained scout dog teams that successfully served in this conflict (2). During the 1960s and 1970s, multiple branches of the military trained dogs for service in the Vietnam War and also for domestic law enforcement and narcotics detection (2). During the Vietnam War, dogs were not only trained as scouts, but also were trained for combat tracking, mine and tunnel detection, and drug detection (2). Due to the expanding roles of these animals, the favored German Shepherd was also joined by the Labrador Retriever, a breed that excelled at detection of, but not physical engagement with, the enemy (2).

After the Vietnam War, scout and combat tracking training ended and law enforcement training expanded to produce what became referred to as “military police working dogs” in the 1977 US Army Field Manual 19–35. In the 1987 US Army Field Manual 19–35, trained canines became referred to as “military working dogs” (MWDs) (2). The MWD family was expanded to include other breeds, such as the Beagle, which were primarily used for detecting drugs and explosives. The Belgium Malinois also joined the MWD program, and broadly excelled at its duties during the Gulf War, the War in Afghanistan, and the Iraq War. In these conflicts, dogs continued to serve as detectors for explosives and narcotics, as well as in search and tracking (4).

In addition to military service, working dogs are employed by law enforcement (“police dogs”, including apprehension dogs, tracking dogs, and detection dogs) and by both search and rescue (“SAR dogs”, including air-scenting, tracking, and trailing dogs) and recovery (cadaver/forensic dogs) professionals.

## Personal Protective Equipment and Decontamination

Working dogs and their handlers form a team and together they encounter the same dangers, including potential exposure to harmful agents. However, unlike their handlers, the canine partner frequently lacks sufficient PPE during an exposure. Limited PPE is available for canines; examples include ear protection (“Mutt Muffs”), eye protection (“Rex Specs” & “Doggles”), paw protection (dog boots) and protective vests (including ballistic vests) (5). PPE for canines is often impractical due to either a lack of tolerance by the animal or because the PPE is an impediment to the performance of the dog's duties. Dog boots may decrease traction or increase the risk of the animal becoming trapped and ear muffs could interfere with communication between the dog and handler (6–8). Coverings designed to protect the respiratory system interfere with the dogs' ability to use its olfactory system (6, 9). Dogs cool themselves by panting; in warm climates or during exertion interference with airflow can contribute to overheating and subsequent heat exhaustion (7).

SAR dogs commonly work in contaminated environments, potentially exposing these animals to a variety of dangerous chemical and biological agents (10–12). MWDs experience similar risks with the additional risk of potential exposure to chemical warfare agents (13). Microbial pathogens, chemical toxins, and chemical warfare agents all present significant risk of harm to both the handler and the canine. PPE and decontamination protocols and kits are readily available for the handler, yet remain problematic for the canine partner. Present-day canine decontamination measures for police and SAR dogs are rarely more sophisticated than shampooing the exposed animal with a degreasing agent (commonly a dishwashing detergent) followed by copious rinsing (11). This protocol is not without logistical challenges, including environmental conditions, supply and equipment availability, the potential need for collection and disposal of contaminated wash and rinse water, and the potential risk of increasing percutaneous absorption via the bathing process (14, 15). MWD decontamination protocols are in some ways more advanced, and are subject to periodic research and development.

Previous studies analyzing potential toxicological hazards to SAR and police canines working disaster sites have described a variety of potential toxicants in multiple forms including solids, liquids, particulates and gases. Solid and liquid chemical agents that are considered potential risks include but are not limited to: hydrocarbons, polychlorinated biphenyls, various toxic metals, soaps, detergents, acids, alkalis, glycols, phenols, alcohols and solvents (9, 12–14, 16). Particulates at disaster sites are numerous and may be either toxic or non-toxic, with some causing little more than acute respiratory and ocular irritation, while others contribute to chronic illness. Gases may be produced by either fires or by chemical reactions. These agents may be hazardous when ingested or inhaled, as well as through dermal or ocular exposure (9, 12). In Otto et al. (17) it is noted that “many potential toxins remain unidentified.”

Long-term risk assessment studies for dogs exposed to agents encountered in disaster sites have been conducted. An epidemiologic investigation of the injuries and illnesses experienced by the dogs (patrol, explosion-detection, and search) working the Oklahoma City bombing was performed. This study revealed that although SAR dogs were 23.6 times more likely to be injured than the explosives detection and patrol dogs, the injuries were minor, with 14 out of 15 injuries occurring to the pads of the paws (18). A study of the morbidity of the SAR dogs on site after the September 11, 2001, terrorist attacks reported an overall low incidence rate for individual morbidities, which included heat exhaustion, fatigue, respiratory difficulty, and numerous superficial cuts and abrasions, mostly of the footpads of the paws (7). An in-depth medical and behavioral survey of the SAR dogs that worked the three major sites associated with the September 11, 2001, terrorist attacks was undertaken. Complete blood counts (CBC) and serum biochemistry profiles were analyzed in deployed dogs and compared to control dogs. In addition, thoracic radiographs were evaluated. The SAR dogs were found to have significantly higher serum globulin concentration which may indicate an increased exposure to antigens at the disaster sites (17). Deployed dogs also had higher bilirubin and alkaline phosphatase activity, which could indicate an increased antigen and toxin exposure (17). There were no pulmonary abnormalities seen in the thoracic radiographs and there was a “lack of clear adverse medical or behavioral effects” seen in the surveyed SAR dogs (17).

A 5 year post-deployment health survey was conducted for the New York Police Department (NYPD) dogs that worked the World Trade Center (WTC) site. Many of these dogs that were deployed to the WTC were on duty throughout the 37-week cleanup operation including the first 3 weeks after the terrorist attack, when fine particulates and noxious gases were at maximum density (10). This study reported that although conjunctival and respiratory tract irritation occurred acutely as a sequela to smoke and particulate exposure, there was no clinical evidence of chronic respiratory disease in these police dogs (10).

In 2010, 23 Federal Emergency Management Agency (FEMA) urban search-and-rescue (USAR) dogs were deployed to Haiti to assist with post-earthquake efforts. An online survey was conducted during a 7–12 month post-deployment period; dehydration was the most common reported condition, followed by wounds, ocular discharge and decreased appetite (6). A 2016 review of the common deployment injuries of urban SAR dogs found the most common documented injury to be abrasions, punctures, and laceration of the pads of the paws, and the second most common medical conditions were dehydration and hyperthermia (commonly referred to as heat fatigue or heat exhaustion) (8).

Although working dogs are more likely to be exposed to chemical contaminants, these dogs may also encounter biological agents that could produce illness. MWDs in particular may encounter biological warfare (BW) agents, including pathogens (bacteria, rickettsiae, and viruses) and toxins (19). Canines are unlikely to be the intended victims during the release of BW agents; additionally, of the BW agents most likely to be encountered by MWDs, the dogs are believed to be less susceptible than the humans to the diseases produced by these agents (13, 14). Decreased susceptibility does not equate immunity and contaminated individuals are a potential hazard to the individuals they come in contact with. Due to a lack of approved canine vaccines for BW agents, administration of prophylactic antibiotics and decontamination with soap and water is considered the standard of care (13). For canines encountering floodwater, bathing with soap and water followed by a dilute hypochlorite (bleach) rinse to neutralize biological contaminants is commonly recommended (14). Bleach is a desirable disinfectant due to its low cost and ease of acquisition; however, other suitable disinfectants are also available (20).

As stated by Perry et al. (21), “evidence-based canine decontamination protocols are underrepresented in the veterinary literature.” Bathing with soap and water is the standard method of gross decontamination for dermal exposures to most toxic substances for both MWDS and SAR dogs (22, 23). High volume, low-pressure water is recommended for working dog decontamination (23). Currently, there are few scientifically investigated low-water field decontamination options available (24).

Since the fur coat potentially delays penetration of agents to the skin, thus delaying cutaneous absorption, effective on-site decontamination of the fur and skin may be carried out before substantial absorption has occurred (23, 24). Delayed absorption is especially significant for MWDs because safe transport of a contaminated canine and enough time to reach a secure decontamination area are not always guaranteed (24). A recent study evaluated a simple field wipe-down procedure in an effort to identify a method that circumvents the need for full-body bathing of working dogs exposed to aerosolized microbiological and chemical contaminants; this study identified disposable towels saturated with 7.5% povidone-iodine scrub as a potential method to decontaminated working dogs (21). Another study evaluating the effectiveness of bathing dogs artificially contaminated with an oil-based pseudo-contaminant noted the need for portable decontamination kits in light of the fact that FEMA SAR canines may be deployed for up to 14 consecutive days and daily bathing may be deleterious to the integrity of the cutis (25).

The most superficial layer of the epidermis is the stratum corneum. The individual cells (squames or keratinocytes) of the stratum corneum are keratinized dead cells and are surrounded by an intercellular lipid derived from lamellar granules produced by the keratinocytes when living (26). The stratum corneum retains moisture and provides a natural barrier against water-soluble chemicals, especially when this layer is cornified (keratinized) (27). The stratum corneum, however, is permeable to fat-soluble (lipophilic) chemicals, which readily pass through the lipid-filled intercellular spaces, ultimately reaching the underlying dermis and passing into the bloodstream via the dermal capillaries (27).

It has been demonstrated in human healthcare workers that frequent hand washing with anionic surfactants damages the barrier function of the skin (15). Sodium lauryl sulfate (SLS) is an anionic surfactant that is a common ingredient in dishwashing detergents as well as both human and pet shampoos. The use of soap and water has been shown to enhance the percutaneous absorption of lipophilic chemicals (15). As pointed out by Buckley and Klingner a goal for skin decontamination in both humans and animals is the use of high molecular weight (MW) solvents in a cleanser that is suitable for a water rinse, the rationale being that chemicals with a MW above 350 are poorly absorbed. Ideally, the cleanser would not defat or otherwise disrupt the natural barrier function of the skin (15).

Lipophilic chemicals penetrate the skin better than water-soluble chemicals; therefore, to increase the penetration of a water-soluble chemical, an oil-based vehicle is used; conversely, the use of water to remove an oil-based chemical can actually cause increased dermal absorption (15). Skin permeability depends on many factors, including the thickness of the skin, the ambient temperature, the hydration of the skin, and the physical condition of the skin. Additionally, contact time, chemical concentration and solubility, and even the vascularity of the skin can each have a contributing role in facilitating the absorption of chemical agents across the skin barrier with subsequent entry into the bloodstream (27).

Historically, providing a barrier to the absorption of chemical agents of war has been a concern since sulfur mustard (HD) was first used by Germany in WWI (26). The US Army produced M-5 protective ointment in 1943–1944, but its issue was discontinued in the 1950s In the 1950s through the 1980s, the US Army Medical Research Institute of Chemical Defense produced two perfluorinated polymer cream blends, one of which progressed through the US Food and Drug Administration as an investigational drug in the 1990s, ultimately becoming approved for use in 2000. This cream became “skin exposure reduction paste against chemical warfare agents” (SERPACWA) and acts as an antipenetrant barrier cream that is effective against both blister and nerve agents. SERPACWA, however, does not neutralize CWAs (27). In 2003, the FDA approved RSDL®, a liquid skin decontamination lotion that neutralizes vesicant chemical or organophosphorus nerve agents (28). RSDL® is currently supplied to all branches of the military for topical human and canine use (13, 29).

Protection of MWDs in a CBRN (“chemical, biological, radiological, nuclear”) environment is a high priority that is acknowledged to be difficult (13). The preferred method of decontaminating MWDs exposed to nerve agents is by first using Reactive Skin Decontamination Lotion (RSDL®), followed by thoroughly washing and rinsing the MWD to ensure all contaminants are removed (13). MWDs exposed to vesicants (blister agents) are decontaminated with thorough washing and rinsing, with the exception of sulfur mustard (H/HD), in which case only RSDL® should be used because “wet skin absorbs more agent than does dry skin”(13). MWD sensitivity to nerve and blister agents varies based on the agent and the exposure form (inhalation vs. dermal exposure); however, in many instances these dogs are less sensitive than their human partner (14). Decreased sensitivity does not equal zero morbidity and contaminated individuals are a potential hazard to the individuals they come in contact with.

Many substances have been evaluated for suitability as decontamination products. These products may be categorized by their mode of action, which includes chemical hydrolysis reactions (acid or alkaline), dry decontaminants, wet decontaminants, and oxidative chemical decontaminants (electrophilic reactions). Chemical hydrolysis reactions that are alkaline effectively neutralize some CWAs. Alkaline pH hypochlorite is an effective hydrolyzer of VX (methylphosphonothioic acid) and G-series nerve agents (27). Dry decontaminants include a wide range of possibilities, such as clean sand, baking or talcum powder, both fuller's and diatomaceous earth, flour, and assorted paper towel products. Dry decontaminants are effective at removing liquid CWAs. M291 SDK was the dry decontaminant kit first issued to US soldiers in 1989. This kit utilizes a resin-based powder in which the “acidic and basic groups in the resin promotes the destruction of trapped chemical agents by acid and base hydrolysis” (27). RSDL® is an example of a wet decontaminant (27). Oxidative chemical decontamination involves the oxidation of sulfur atoms in VX and HD (sulfur mustard) by positively charged chloride ions in chlorine chemicals. 0.5% solutions of either sodium or calcium hypochlorite are effective for decontamination of skin via oxidative chlorination, therefore “plastic bottles containing 6 ounces of calcium hypochlorite crystals are currently fielded for this purpose” (27).

Working dogs are not only valued for their contributions to urban disasters and war, but are also monetarily highly valuable. At present, there are over 2,500 active US MWDs in the field, each with a dog handler who has completed a minimum 17-week Advanced Individual Training program (30, 31). A single fully-trained US bomb detection MWD is estimated to be worth approximately $150,000 (32). The Search Dog Foundation estimated it costs on average roughly $41,000 to create a Certified Search Team (33).

In summary, the use of PPE for canines is often impractical; shampooing with copious rinsing is not without logistical challenges, may increase percutaneous absorption, and should be limited in frequency; and current decontamination products were developed for humans. It is logical to assume that evidence-based canine decontamination protocols are underrepresented in the veterinary literature (21) because they are underrepresented in research and development. Thus, further research is warranted to establish proven chemical class-specific treatment protocols that will effectively decontaminate working dogs following exposures in the field, protecting not only the dogs but their handlers.

## Author Contributions

RG conceptualized, led weekly meetings to discuss information finding and writing progress, supervised MB who co-wrote the first draft, and final review of manuscript. SH co-led weekly progress meetings, co-supervised MB who co-wrote the first draft, and critiqued and modified first draft before sending to RG. CJ attended weekly progress meetings and co-wrote the first draft with MB. MB attended weekly progress meetings and co-wrote the first draft with CJ. All authors contributed to the article and approved the submitted version.

## Funding

This study was supported by US Department of Defense, SBIR Phase II Contract W911NF-21-C-004. We wrote the present review after realizing there was limited published information for us to use relative to the funding. The DOD funding paid for the summer veterinary student who largely prepared the first draft of our report. However, the DOD had no stated interest that we publish such a report.

## Conflict of Interest

The authors declare that the research was conducted in the absence of any commercial or financial relationships that could be construed as a potential conflict of interest.

## Publisher's Note

All claims expressed in this article are solely those of the authors and do not necessarily represent those of their affiliated organizations, or those of the publisher, the editors and the reviewers. Any product that may be evaluated in this article, or claim that may be made by its manufacturer, is not guaranteed or endorsed by the publisher.
